# Levels and health risk assessments of Phthalate acid esters in indoor dust of some microenvironments within Ikeja and Ota, Nigeria

**DOI:** 10.1038/s41598-023-38062-4

**Published:** 2023-07-11

**Authors:** Winifred U. Anake, Esther A. Nnamani

**Affiliations:** Department of Chemistry, College of Science and Technology, Covenant University, P.M. B 1023, Ota, Ogun State Nigeria

**Keywords:** Environmental chemistry, Environmental sciences

## Abstract

The levels, profiles of Phthalate acid esters (PAEs) and their associated health risk in children and adults using indoor dust samples were assessed from nine (9) microenvironments in Nigeria. Six PAEs congeners were determined using Gas Chromatography–Mass Spectrometry and the human health risk assessments of PAEs exposure to children and adults were computed using the United States Environmental Protection Agency (USEPA) exposure model. The mean concentrations of the total PAEs (Σ_6_PAEs) in indoor dust across the study locations ranged from 1.61 ± 0.12 to 53.3 ± 5.27 μg/g with 72.0% of di-*n*-octyl phthalate (D*n*OP) as the most predominant contributor of PAEs in sample locations B, C, D, E, F and G. PAEs estimated daily intake results exceeded the USEPA value of 20 and 50 kg/bw/day for children and adults respectively in some locations. Non-carcinogenic risk exposure indicated no risk (HI < 1), while the carcinogenic risk was within the recommended threshold of 1.00 × 10^–4^ to 1.00 × 10^–6^ for benzyl butyl phthalate and bis-2-ethylhexyl phthalate. From our findings, lower levels of PAEs were observed in locations with good ventilation system. Also, the human health risk evaluation indicated indoor dust ingestion as the dominant exposure route of PAEs for both children and adults, while the children were at a higher risk of PAEs exposure. To protect children susceptible to these endocrine-disrupting pollutants, soft vinyl children’s toys and teething rings should be avoided. Appropriate policies and procedures on the reduction of PAEs exposure to humans should be enacted by all stakeholders, including government regulatory agencies, industries, school administrators and the entire community.

## Introduction

There is a growing concern about environmental chemical exposure and its adverse effects on human physiology across the globe^1,2^. The indoor environment is becoming a major concern because people spend about 90% of their time indoors^3,4^. This environmental risk factor is critical to the susceptible populace, such as infants and children^1,5–7^. The outcome of this environmental risk factor is pronounced more during their sensitive periods of growth^8,9^. This is because their dose intake is higher compared to adults due to their increased breathing rate per unit of body weight^10–13^. Also, children growing immunological and physiological functions make them more vulnerable to these chemical toxins^3,10,14^. Moreover, ambient air affects indoor air quality, and it has been scientifically validated that pollutants concentration is more in indoor air than in an outdoor environment^7,15^. This is due to several indoor sources, behavioural patterns, cleaning habits and building characteristics^16,17^. Consequently, the United Nations Sustainable Development Goal (SDG) 3.9 continues to address this issue as it seeks to minimize the number of illnesses and deaths resulting from toxic chemicals in the environment.

Phthalate acid esters (PAEs) are a group of chemicals with a basic structure characterized by phthalic acid with alkyl groups’ alcohol through an ester bond. PAEs are one of the major emerging pollutants of critical concern^18,19^. They are also endocrine-disrupting chemicals (EDCs). EDCs are chemical compounds that interfere with the endocrine system in humans and trigger cancerous cells, birth defects, and developmental disorders^19,20^. Although regulatory limits for a few chemical pollutants have been established due to the harm they cause to the human body, endocrine-disrupting chemicals such as PAEs are still widely used in a diversity of consumer and household items^21^. PAEs are also known as semi-volatile organic compounds (SVOCs). SVOCs have been found in indoor environments, and they have been proven to be about two to five times more prevalent than those present in outdoor environments^22,23^. PAEs are known to be ubiquitous in indoor environments due to their primary use as plasticizers and solvent carriers in diverse industrial applications^2,3,19,24^.

Dust ingestion is children’s most common exposure route to this indoor chemical pollutants^3^. This is because of their frequent hand-to-mouth activities^25^. Previous reports have revealed that indoor dust on the impervious external layer serves as a reservoir for organic pollutants^26,27^. Therefore, it can be inferred that indoor dust is a major source of indoor environmental pollutants such as PAEs^28^. It is also an important entry point for many chemical contaminants in young children^29^. The existence and fate of PAEs in an indoor environment are attributed to leachability, indoor air volume, rate of air exchange between the indoor and outdoor environments, humidity, building features and indoor temperature^11,30^. The study of PAEs in indoor dust commenced in 1997. PAEs have been accessed globally in different microenvironments such as daycare centres, preschool centres and elementary schools, workplaces, dormitories and homes^11,12,16,17,21,28,31^. Table 1 briefly describes the six predominant PAEs congeners (Di-2-ethylhexyl phthalate (DEHP), Di-n-butyl phthalate (DBP), Di-*n*-octyl phthalate (D*n*OP), Butyl benzyl phthalate (BBP), Di-ethyl phthalate (DEP), and Di-methyl phthalate (DMP)), their uses, and product derivatives.

**Table 1:** Description of predominant PAEs.

Phthalate compound	Molecular weight (g/mol)	Use	Product
DEHP	390.56	Plasticizer for PVCs	Baby dolls, shoes, raincoats, clothing, medical devices, furniture, automobile upholstery, and floor tiles
DBP	278.35	Plasticizer for PVC, PVA, and rubber. Manufacture of solvent and preservatives in paint and cosmetic materials	Latex adhesives, sealants, car care accessories, cosmetic products, ink and dye materials, insecticides, herbicides and pesticides, food wrapping materials, furniture and upholstery, and pharmaceutical accessories
DNOP	390.56	Plasticizer for PVCs	Floorings, tarpaulins, pool liners, holes, and pipes
BBP	312.37	PVCs Plasticizer, polyurethane, polysulfide, and acrylic-based polymers	Poly-vinyl carpets, silicone sealants and adhesives, car care accessories, food wrapping material, and artificial leather materials
DEP	222.24	Cosmetics and perfumes	Skin-care products and fragrances
DMP	194.19	Cosmetics and fragrance	Skin-care products and fragrances

^11,12,32,35,36^.

Many investigations have ascertained the relationship between PAEs exposure and their associated human health risk predominantly in their reproductive and growth pattern^33,34^. Exposure to PAEs in children may lead to a high risk of atopic disorder^35^. They may also cause reproductive health problems, particularly in males^36,37^. They can also lead to premature development of the breast and loss of pregnancy in females^11,38^. Also, a significant relationship has been reported to exist between asthma and allergic signs due to PAEs exposure in preschool children^25,38–40^. There are also reported cases of obesity due to PAEs exposure^33,41^. Reports from Denmark^42^, Sweden^42,43^, Germany^44^, USA and China^2,3,39,45–47^ reveal that the sampled locations were contaminated with PAEs. Also, many epidemiological studies have shown that PAEs exposure to indoor dust has been linked to diverse reproductive effects, acute neurological disorders, inflammations and allergies^36,37,48^. Several studies on PAEs have been conducted in Nigeria, using water, food materials, PET bottles, breast milk and urine samples except for dust as environmental monitoring tools^49–54^. Hence, studying PAEs levels and the degree of exposure via the three predominant routes of indoor dust in different microenvironments is important. Therefore, the present study aimed to (1) determine the levels and profile of PAEs in indoor dust from nine (9) different microenvironments in Ikeja and Ota, Nigeria; and (2) assess the predominant route of PAEs exposure and evaluate the human health risk assessment for children and adults. To the best of our knowledge, this is the first study to report the levels and human health risk evaluation of PAEs in indoor dust from daycare centres in Nigeria and in Africa.

## Materials and methods

### Chemicals and solvents

PAEs mix standard inclusive of butyl benzoyl phthalate (BBP), di ethyl phthalate (DEP), di methyl phthalate (DMP), di butyl phthalate (DBP), di-*n*-octyly phthalate (D*n*OP), and bis-2-ethylhexyl phthalate (DEHP) along with benzyl benzoate (BB) internal standard was purchased from AccuStandard, USA, Inc. Analytical grade n-hexane was purchased from Merck, Germany while analytical grade acetone and methanol were purchased from Fisher Scientific, United Kingdom.

### Description of the study area

**Figure 1:**
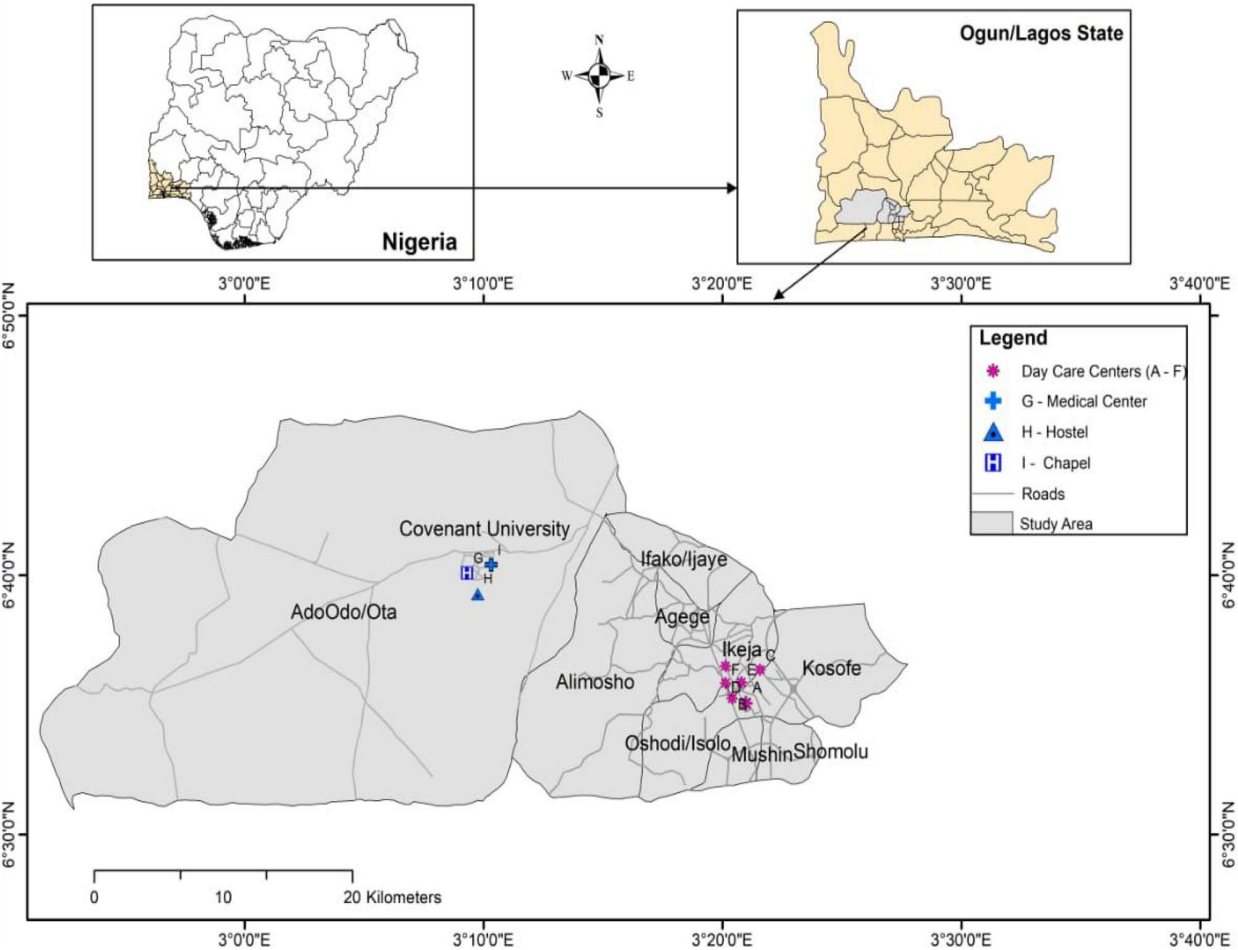
Map of the study areas. The map was generated from geospatial analysis of ArcGIS desktop software (version 10.3).

Indoor dust samples were collected from nine microenvironments in the Nigerian cities of Ikeja and Ota, as depicted in Fig. 1 and Table S1. Ikeja is the capital and commercial heart of Nigeria, while Ota is known for major industrial activities, as many manufacturing industries in Nigeria have their factories there. Ikeja and Ota are located at 6° 31′ 46″ N; 3° 21′ 48″ E 78.2 km and 6° 39′ 27″ N; 3° 11′ 18″ E 78.2 km, respectively. The different microenvironments were assigned sample location codes A to I. Location A–F (preschools), G (hospital: pediatric section), H (hostel) and I (chapel). The locations were sampled for two months (December 2021–January 2022).

### Sample collection and preparation

Indoor dust particles were sucked from the sample locations using Tesco vacuum cleaner VCBL17. The vacuum brush was washed with methanol before and after each sampling to avoid cross-contamination of the samples^2–4^. Nine different indoor microenvironments were sampled twice during the dry season (December 2021–January 2022). In each of the locations, dust samples were collected every two hours throughout the day from different sections and a composite sample for each location was used for the analysis. A total of nine indoor dust samples and six control samples were collected. After sampling, the dust particles were transferred to aluminium foil and sealed in a ziploc bag, then transferred to the laboratory^12^. To remove unwanted substances and homogenize the sample, the collected dust samples were sieved with individual < 150 µm mesh and wrapped in aluminium foil, then stored in an airtight ziploc bag in the freezer at − 20 °C prior to further analysis^2–4,12^.

### Sample extraction and clean up

Dust sample (100 mg) were measured with an analytical weighing balance and quantitatively transferred to a 10 mL glass tube. Exactly 3 mL of the extraction solvent (hexane and acetone) in the ratio of 1:1 was introduced into the glass and placed on the vortex mixer for 20 min at 1500 rotation per minute (rpm) to re-suspend and agitate the mixture. Afterwards, the agitated mixture was centrifuged for 20 min at 1500 rpm. This cycle was repeated two more times for better extraction efficiency, and the pool of extract was concentrated to 1 mL with the aid of a rotary evaporator and reconstituted with 2 mL of methanol^5,12^. After re-solubilisation, clean—up of samples was performed on a 30 mL glass chromatographic column packed with 10 g of activated silica gel (60–200 mesh size), and 1 g of activated anhydrous sodium sulphate (Na_2_SO_4_) was introduced into the packed column for absorption of moisture. The packed column was pre-eluted with n-hexane at a 2 mL/min flow rate. The extract was carefully transferred into the column, and 2 mL of n-hexane was used to thoroughly rinse and transfer the extract down the column. The column was eluted with 15 mL of n-hexane, allowing the non-polar fraction of the extract in the column to be collected and discarded. After that, 20 mL of acetone was added to the column to elute the targeted analyte. The eluent was collected and transferred to a 100 mL round bottom flask and pre-concentrated to 1 mL with a rotary evaporator. The concentrated extract was rinsed with 2 mL of methanol before being transferred to an aluminium-wrapped GC glass vial for instrumental analysis. This sample preparation and extraction methods are consistent with the USEPA 8061A method for PAEs analysis.

### Instrumental analysis of PAEs using GC–MS

The extracts were analyzed with the use of Agilent J&W Gas chromatography–mass spectrometry coupled to an electron ionization (EI) source. Agilent J&W HP 5 Capillary column having a 30 m × 0.25 mm dimension of silica and a 0.25 μm thickness was used for the isolation. The single ion monitoring mode was used to quantify each target compound. The carrier gas, 99.9% pure ultra-high helium gas, was kept at a flow rate of 1.2 mL/min. The column temperature program began at 100 °C, was held for 1 min, and then ramped to 280 °C at a rate of 20 °C per minute for 7 min. The solvent delay lasted for 3 min. The temperatures of the ion source, injection port, quadruple, and transmission line were kept at 230 °C and 250 °C, respectively. An injection volume of 1 µL and an analysis time of 17 min was used for the six PAEs compounds^12,16,55^.

### Human health risk assessments estimation

PAEs have been linked to serious health consequences in humans^56^. PAEs non-carcinogenic and carcinogenic human risk assessment for both children and adults in this study were evaluated by the USEPA 2011 model with a minor modification^57^. Based on the single PAEs congeners, DMP, DEP, DBP, and D*n*OP have been identified as non-carcinogenic, while BBP and DEHP are known to be carcinogenic^3^. To calculate the estimated daily intake (EDI) of PAEs through dust ingestion, Eq. (1) was used. The formulae outlined in Eqs. (2) to (4) were used to examine the average daily dose (ADD mg/kg^/^d) for children and adults through the different phthalate exposure pathways, namely; ingestion, dermal and inhalation^17,29,56,58^. These formulae have been extensively used in previous studies^2,3,17,59,60^.

1 $$EDI = C_{dust} \times {\raise0.7ex\hbox{$f$} \!\mathord{\left/ {\vphantom {f m}}\right.\kern-0pt} \!\lower0.7ex\hbox{$m$}}$$

C_dust_ = concentration of PAEs compound in dust particles (µg/g); f = ingestion rate of dust (g /day); m = body mass (kg)

2 $$ADD_{ing} = \frac{{C_{dust} \times IR_{ing} \times EF \times ED \times CF}}{BW \times AT}$$

3 $$ADD_{der} = \frac{{C_{dust} \times SA \times AF \times ABF \times EF \times ED \times CF}}{BW \times AT}$$

4 $$ADD_{inh} = \frac{{C_{dust} \times IR_{inh} \times EF \times ED}}{BW \times AT \times PEF}$$

IR_inhalation_ = dust inhalation; SA = area of dermal exposure; ABF = fraction of dermal absorption; AF = dust dermal adherence factor; BW = body mass index expressed in kilogram; AT = average time (d); AT = ED × 365 (non-cancer risk); AT = LT $\times$ 365 (cancer risk); ED = exposure period (y); EF = exposure frequency (d/y); CF = conversion factor (1.0 $\times$ 10^–6^ kg/mg); PEF = particle emission factor (1.36 $\times$ 10^−9^ m^3^/kg); The hazard quotient (HQ) and hazard index (HI) was used to calculate the non-carcinogenic risk of indoor dust PAEs exposure as shown in Eqs. (5) and (6);

5 $$HQ_{i} = \frac{{ADD_{i} }}{RfD}$$

6 $$HI = \sum {HQ_{i} }$$

where RfD is the independent phthalate compound reference dose value (mg/kg/d). The reference dose is defined as the maximum daily risk that can be imposed on the exposed population^60^. It is used as an indicator or measure to indicate the possibility of a serious health effect over the course of a person’s life. While HQ signifies the health risk of each PAE to humans through the diverse exposure routes, and i signifies the various exposure routes (dermal absorption, ingestion and inhalation). HI value greater than 1 indicates that adults and children exposure to PAEs might result in a non-cancer risk. DMP, DEP, DBP, and D*n*OP are non-carcinogenic, whereas DEHP and BBP are carcinogenic to humans. The carcinogenic route was evaluated using the lifetime average daily exposure doses (mg/kg/d) shown in Eqs. (7) and (9)

7 $$LADD_{der} = \frac{{C_{dust} \times ABF \times CF}}{AT}\left( {\frac{{SA_{child} \times AF_{child} \times ED_{child} \times EF_{child} }}{{BW_{child} }} + \frac{{SA_{adult} \times AF_{adult} \times ED_{adult} \times EF_{adult} }}{{BW_{adult} }}} \right)$$

8 $$LADD_{inh} = \frac{{C_{dust} }}{AT \times PEF}\left( {\frac{{IR_{inhalation} \times ED_{child} \times EF_{child} }}{{BW_{child} }} + \frac{{IR_{inhalation} \times ED_{adult} \times EF_{adult} }}{{BW_{adult} }}} \right)$$

9 $$LADD_{ing} = \frac{{C_{dust \times CF} }}{AT}\left( {\frac{{IR_{ingestionchild} \times ED_{child} \times EF_{child} }}{{BW_{child} }} + \frac{{IR_{ingestionadult} \times ED_{adult} \times EF_{adult} }}{{BW_{adult} }}} \right)$$

The carcinogenic risk (CR) was analyzed using Eq. (10)

10 $$CR = \,LADD \times CSF$$

CSF stands for cancer slope factor, inclusive of CSF_oral_, CSF_ingestion_, CSF_inhalation_, and CSF_dermal_. It is a measure of the likelihood or possibility of an individual being infected by cancer due to oral intake of the chemical^61^. To calculate the possibility of cancer risk via inhalation and dermal absorption exposure pathway, it was estimated that CSF_inhalation_ and CSF_dermal_ are equal to CSF_oral_^10^. The evaluated values from cancer risk calculation are considered negligible if CR is below 10^–6^. The permissible limit is 10^–6^ to 10^–4^, while values higher than 10^–4^ are considered a threat to health^3,17^. Table 2 shows the parameters used to calculate the human risk assessment.

**Table 2:** Human health risk assessment and exposure parameters.

Parameters	Unit	Child	Adult
Concentration (C)	µg/g	–	–
IR_ingestion_	mg/d	200	100
IR_inhalation_	m3﻿/d	5	20
EF	d/y	330	304
ED	Y	6	24
CF	kg/mg	1.0 × 10^−6^	1.0 × 10^−6^
BW	Kg	15	60
AT	Days	365 × ED	365 × ED
AT (for non-cancer risk)	D	ED × 365	–
AT (for carcinogenic risk)	D	LT × 365	–
SA	cm^2^	1700	4000
AF	mg/cm^2^/d	0.2	0.07
ABS	–	0.001	0.001
PEF	m^3^/kg	1.36 × 10^–9^	1.36 × 10^–9^
CFS_oral_	kg/d/mg	BBP	0.0019
		DEHP	0.014
RfD	mg/kg/d	DMP	10
		DEP	0.8
		DBP	0.1
		BBP	0.2
		DEHP	0.02
		D*n*OP	0.4
LT	Y	54.44	54.20

^11,12,59,60^.

### Quality assurance/quality control

The suitability of the analytical and instrumental processes were ensured by avoiding all forms of plastics throughout the laboratory procedures. The glassware used for the work was thoroughly oven-baked for 6 h at 120 °C in line with the USEPA 8061A method for the treatment of glassware and rinsed with acetone and hexane thereafter. The instrumental system was calibrated by preparing an equal volume of the external and internal standards in 5 mL methanol. A five-point calibration curve was plotted between the areas of peak and calibration concentrations resulting in an overall linearity range of 0.996–1 for the six PAEs compounds. To test the suitability of each of the analytical procedures, blank samples were prepared, namely, field blank, sieve blank, method blank, and reagent blank. The six phthalate compounds for the study were found in the blank samples and deducted from the sample concentrations to obtain the exact value of PAEs in the sample set. The method precision from the duplicate blank was 11.14%. This value is within the expected range of < 20% and is consistent with other reported works on PAEs in indoor dust particles such as^2,12,59,62^. The recovery study was done to ascertain the extraction efficiency, and the value ranged from 70.9 to 72.6%. This range is in agreement with the expected range of 70–120% for PAEs recovery study in indoor dust particles^12,29^. The computation of the limit of detection (LOD) and limit of quantification (LOQ) was done using a signal-to-noise ratio of 3:1 and 10:1, respectively. The LODs and LOQ of PAEs ranged from 0.005 (DEP) to 0.025 (DBP) and 0.016 (DEP) to 0.083 (D*n*OP), respectively, as shown in Table S2.

### Statistical analysis

Descriptive statistics were done using Microsoft Excel 2013 and Origin Pro 8.5. The Shapiro–Wilk test was applied to check the normality of the data. The data were not normally distributed as such, the non-parametric Kruskal–Wallis test was used to compare PAEs concentration differences between the various microenvironments. A *p-value* < 0.05 was considered significant.

## Results and discussion

### Concentrations of PAEs in indoor dust

**Figure 2:**
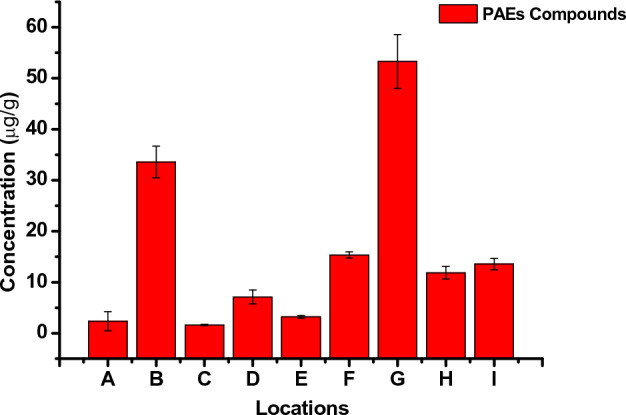
Concentration of total PAEs compound in each location.

The concentrations and detection frequency of PAEs from the sampled sites (Ikeja and Ota) are shown in Figure S1. There was a 100% detection frequency for the six PAEs compounds. The concentrations of total PAEs compound in each location are shown in Fig. 2 and Table S3. D*n*OP was the most abundantly detected compound in all nine samples, followed by DMP and DBP. The mean concentrations of the six PAEs compound in indoor dust ranged from 1.61 ± 0.12 to 53.3 ± 5.27 μg/g across the different microenvironments. There were variations in the concentration of PAEs in indoor dust across the sampled locations. An overview of the study locations' indoor characteristics and materials is shown in Table S1. Materials found in indoor environments like medical devices in the children’s ward in a hospital, floor tiles, wall covering decorative items, chairs, tables, children’s play toys and articles in preschool centres, television sets, adhesives, wires and cables have D*n*OP as the major plasticizers used in their production, which led to D*n*OP being the dominant pollutants found in seven out of the nine sampled locations. The highest level of Σ_6_PAEs was found in indoor dust from location G representing a paediatrics’ ward in the hospital, with a mean value of 53.3 ± 5.27 μg/g, followed by two preschool centres in locations B and F with mean concentrations of 33.6 ± 3.10 and 15.3 ± 0.62 μg/g. The high level of PAEs in these locations is attributed to inadequate ventilation as a result of little or no exposure to sunlight because the window blinds were permanently closed, thereby preventing the free flow of air and the penetration of sunlight^63,64^. Also, Liao et al.^65^ findings observed reduced levels of indoor PAEs, using mechanical ventilation and air cleaner than with only natural ventilation. Hence, they recommended the use of mechanical ventilation or natural ventilation along with air cleaners to reduce the concentration of PAEs indoors.

The lowest Σ_6_PAEs concentrations were from locations A (2.34 ± 1.86 μg/g) and C (1.61 ± 0.12 μg/g) day-care centres in Ikeja, Lagos, Nigeria. Both locations were observed as the most ventilated and spacious locations among all the microenvironments. Furthermore, levels of total PAEs reported in this work were lower than those reported in other regions, such as Greece 797 μg/g^66^, China 592 μg/g^12^, Saudi Arabia 663 μg/g^10^, and USA 214 μg/g^55^. The gas/particle partitioning of PAEs is integral to their indoor fate and health risks. However, the effects of indoor environmental parameters such as temperature, humidity and the partitioning of PAEs between air and particles is rarely known^64,67^. In addition, it is worth stating that the particle-air partition coefficient and vapour pressure properties affect the existence of PAEs congeners in both phases of individual PAEs. The equilibrium typically favours the gas phase for PAEs with higher vapour pressure and a lower particle air coefficient, while the particle phase is in parity with PAEs with lower vapour pressure and a higher particle air coefficient^29,55^. The high concentration of DEP and DBP in the hostel and chapel indoor dust might be connected to their solvent-carrying properties in the production of several household and skin care materials such as body lotion and deodorants, insecticides and pesticides, plastics and sealants during production in spite of the high volatility of DEP and their reported existence predominantly in the gas phase than dust phase^12^. The location with the highest concentration of DEHP was found in a preschool centre with PVC flooring. This result is in conformity with studies carried out by^12,29,68,69^. As they all found a significant correlation between PVC flooring and the concentration of DEHP in indoor dust. The variation in the mode of sampling, extraction and clean-up procedures, climatic factors, the lifestyle of the occupants, indoor maintenance, flooring type, building characteristics, and decorative wall items are all factors contributing to the levels of PAEs in indoor dust^11,29,68–70^. Apart from different analytical procedures, the lack of inter-laboratory comparisons of PAEs results in dust and the inability to analyze with standard reference material makes the comparison of results difficult. Consequently, a high variation was observed between concentrations determined in different study locations and similar neighborhoods in the same country.

One-way ANOVA of the parametric method could not be used since the data were not normally distributed^58,71,72^. Hence, the Kruskal–Wallis test of the non-parametric method was used to compare PAEs concentration differences between the various microenvironments. The result presented in Table 3 showed that there was a statistically significant difference between PAEs compounds and microenvironments since the *p-value* (0.001) < 0.05 level of significance. Kruskal–Wallis’ mean rank for each of the PAEs compounds is presented in Table 4.

**Table 3:** Kruskal–Wallis test.

Test statistics^a,b^	
	PAEs Scores
Kruskal–Wallis H	22.842
df	5
Asymp. Sig.	0.001

^a^Kruskal Wallis test.

^b^Grouping variable: PAEs compound.

**Table 4:** Kruskal–Wallis mean ranks for each of the PAEs compound.

	PAEs compounds	N	Mean rank
PAEs Scores	DMP	9	19.11
	DEP	9	26.44
	DBP	9	36.56
	BBP	9	21.11
	D*n*OP	9	45.22
	DEHP	9	16.56
	Total	54	

The Σ_6_PAEs concentrations in indoor dust from selected reports are presented in Table 5. Globally, PAEs are among the identified indoor chemical pollutants that thrive in children learning environments^69^. DEHP, DBP, DEP, and DMP were the most commonly reported across regions of the world, with DEHP being the highest PAEs compound detected across different countries studied. The trend of reported results in decreasing order include: South Korea > China > Denmark > Sweden > USA > Saudi Arabia > Nigeria. This study reported a different trend of result because D*n*OP was the most predominantly detected PAEs compound and there are a few other reported studies with similar trends of result^16,42^. As indicated in Table 5, the highest DEHP concentration of 3030 ug/g reported in South Korea far outweighs the 0.018–1.41 ug/g concentration obtained in this study. Since this is the first study of PAEs in indoor dust on the shores of Africa, there was no basis for comparison within the continent.

**Table 5:** Concentrations (μg/g) of six PAEs in indoor dust from selected reports.

Country	Sample size	Environment	DMP	DEP	DBP	BBP	DEHP	D*n*OP	References
Nigeria	9	Daycare centres, hospital, hostel and chapel	0.074–0.400	0.023–8.86	0.12–5.06	0.068–0.620	0.018–1.410	0.363–46.09	This study
Denmark	500	Children homes	NS	1.7	15	3.7	210	NS	^41^
	151	Daycare centres	NS	2.2	38	17	500	NS	
South Korea	82	Daycare centres	NS	375 (66–556)	107 (29–908)	336(< LOD-1369)	358 (222–673)	NS	^73^
	79	Kindergartens	NS	52.8 (< LOD-170	117 (31–719)	333(< LOD-1422)	375 (66–556)	NS	
South Korea	50	Nursery schools	2.1	–	52.0	50.4	3030	–	^69^
China	14	Offices	–	NS	135	NS	581.5	–	^45^
USA (geometric mean)	39	Early child education centres	NS	1.7	18.2	68.8	179.5	NS	^74^
Sweden	100	Preschools	0.1	0.46	6.4	8.7	290	NS	^75^
China	5	Kindergartens	NS	ND	31.2	NS	202	ND	^3^
	10	Offices	NS	ND	145	NS	1310	ND	
Saudi Arabia (median–min–max)	10	Offices	ND	28 (25–45)	175 (100–260)	ND	110 (90–125)	NS	^59^

NS, not studied; ND, not detected.

### Indoor dust composition profiles of Phthalate acid esters from different microenvironments

**Figure 3:**
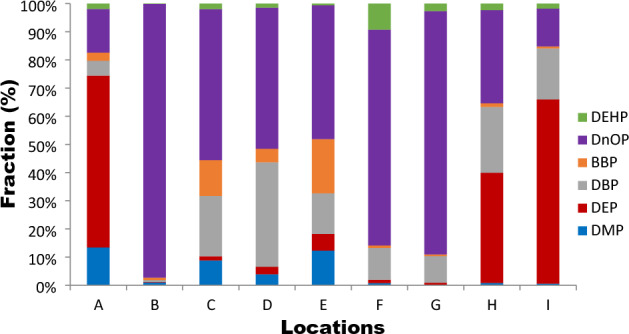
Composition profiles of PAEs in indoor dust.

The composition profile of PAEs from indoor dust across sample locations A to I are shown in Fig. 3. The first three most abundant PAEs congeners of the Σ6PAEs, in indoor dust with associated proportions and concentrations respectively are as follows: Location A: DEP (1.04%, 1.43 ug/g), D*n*OP (15.3%, 0.36 ug/g), and DMP (13.4%, 0.31 ug/g); Location B: D*n*OP (97.2%, 32.7 ug/g), DMP (1.07%, 0.33 ug/g) and BBP: 0.84%, (0.29 ug/g); Location C: D*n*OP (53.1%, 0.86 ug/g), DBP(21.5%, 0.34 ug/g) and BBP (12.8%, 0.20 ug/g); Location D: D*n*OP (49.5%, 3.57 ug/g), DBP(35.7%, 2.64 ug/g) and BBP (4.76%, 0.34 ug/g); Location E: D*n*OP (47.6%, 1.53 ug/g), BBP(19.3%, 0.62 ug/g) and DBP (14.4%, 0.46 ug/g); Location F: D*n*OP (76.7%, 11.76 ug/g), DBP(11.3%, 1.74 ug/g) and DEHP (9.2%, 1.41 ug/g); Location G: D*n*OP (86.5%, 46.1 ug/g), DBP(9.50%, 5.06 ug/g) and DEHP (2.63%, 1.40 ug/g); Location H: DEP (39.2%, 4.65 ug/g), D*n*OP (33.1%, 3.93 ug/g) and DBP (23.4%, 2.80 ug/g); Location I: DEP (65.4%, 8.86 ug/g), DBP (18.2%, 2.46 ug/g) and D*n*OP (13.4%, 1.82 ug/g). The composition profiles of PAEs from the nine sampled locations show similarities in the first three most abundant PAEs congeners between locations C and D (D*n*OP  > DBP > BBP) and locations F and G (D*n*OP  > DBP > DEHP), respectively. This similar trend of PAEs composition profile indicates a common source of those specific PAEs in the indoor microenvironment and agrees with previously reported studies^2,55,73,74,76^. Other sample locations indicated variable PAEs congeners’ trends in the composition profiles. This is a pointer to the variation in the sources in the different indoor microenvironments. This finding is similar to that of Liu et al^77^, who opined that the variation in indoor pollution sources for different indoor spaces makes the mass transfer of PAEs in such environment more complicated. Also, six (6) locations recorded D*n*OP as the most predominant contributor of PAEs, except for locations A, H and I, where DEP was the most abundant contributor (61.0, 39.2 and 65.4%, respectively). The highest contributions of D*n*OP and DEP could be linked with plasticizers and personal care products containing D*n*OP and DEP in the indoor environment^10,67^. In addition, the result of a chamber study on the migration of D*n*OP between source surfaces and settled dust by Li et al^78^ is a pointer to the fact that direct contact contributes significantly to the migration of D*n*OP from the sources to the dust.

### Human health risk estimation

Tables 6 and 7 present the results of the human health risk assessments for this study. Table 6 reveals the estimated daily intake (EDI) of total PAEs in each microenvironment. Location G representing the children's ward in the hospital, had the highest concentration of EDI, 691 and 86.4 μg/g, respectively, for both children and adults. Exception of locations A and C, individual PAEs compounds recorded in other investigated locations (B, D, E, F, G, H, and I) were above the threshold of 20 kg /bw/day for children. Furthermore, the EDI for adults were above the stipulated limit of 50 kg /bw/day as estimated by USEPA in location B and G. This indicates that the daily exposure to PAEs compounds in these locations was very high, thus harmful to the occupants of the environment.

**Table 6:** Human health risk exposure to PAEs in indoor dust in different microenvironments.

Parameters	Human	A	B	C	D	E	F	G	H	I
DI	Children	11.63	428.12	1.86	75.33	23.34	185.03	691.27	138.64	161.16
	Adult	1.45	53.52	0.23	9.42	2.92	23.13	86.41	17.33	20.14
ADD_ing_	Children	1.05E−05	3.87E−04	1.67E−06	6.81E−05	2.11E−05	1.67E−04	6.24E−04	1.25E−04	1.45E−04
	Adult	1.21E−06	4.45E−05	1.93E−07	7.84E−06	2.43E−06	1.93E−05	7.19E−05	1.44E−05	1.67E−05
ADD_der_	Children	1.78E−08	6.58E−07	2.85E−09	1.16E−07	3.59E−08	2.84E−07	1.06E−06	2.13E−07	2.47E−07
	Adults	3.39E−09	1.24E−07	5.41E−10	2.19E−08	6.80E−09	5.39E−08	2.01E−07	4.04E−08	4.70E−08
ADDi_nh_	Children	7.73E−09	2.84E−07	1.23E−09	5.01E−08	1.55E−08	1.23E−07	4.60E−07	9.22E−08	1.07E−07
	Adults	8.91E−10	3.28E−08	1.42E−10	5.77E−09	1.79E−09	1.42E−08	5.29E−08	1.06E−08	1.23E−08
HI	Children	2.56E−09	9.07E−04	3.85E−06	4.14E−04	6.49E−05	1.33E−03	2.79E−02	2.67E−03	1.89E−03
	Adults	2.96E−07	4.20E−05	1.76E−06	4.80E−05	8.92E−06	1.54E−04	3.20E−04	3.08E−04	2.18E−04

**Table 7:** Human health risk exposure.

		Non-carcinogenic intake				Carcinogenic intake		Total intake
Parameters	Human	DMP	DEP	DBP	D*n*OP	BBP	DEHP	Total
DI	Children	13.16	190.41	180.11	1122.76	11.621	24.20	1542.26
	Adults	1.31	23.69	22.41	156.38	1.971	5.30	211.06
ADD_ing_	Children	1.15E−05	1.72E−04	2.67E−04	5.61E−04	4.21E−05	6.14E−04	1.67E−03
	Adults	1.17E−06	1.98E−05	3.09E−05	1.03E−04	4.94E−06	9.66E−06	1.69E−04
ADD_der_	Children	2.02E−01	1.27E−01	2.77E−07	1.92E−06	6.45E−03	6.51E−08	3.35E-01
	Adults	3.84E−09	5.55E−08	5.25E−08	3.65E−07	4.62E−09	4.98E−08	5.31E−07
ADD_inh_	Children	8.75E-09	1.27E−07	1.20E−07	8.32E−07	1.05E−08	2.82E−08	1.13E−06
	Adults	1.01E−09	4.16E−08	2.05E−08	9.90E−08	1.21E−09	3.81E−09	1.67E-07
HQ_ing_	Children	1.15E−06	2.15E−04	2.67E−03	1.40E-03	2.10E−04	3.07E−02	3.52E−02
	Adults	1.33E-07	2.05E−04	1.89E−03	6.09E−04	1.58E-04	3.26E−06	2.87E−03
HQ_der_	Children	2.02E-09	3.66E−07	2.77E−06	4.81E-06	1.22E−07	3.26E−06	1.13E-05
	Adults	4.09E−10	3.50E−07	1.54E−07	1.54E−06	2.73E−08	2.09E-06	4.16E−06
HQ_inh_	Children	8.75E−10	1.58E−07	1.20E−06	2.08E-06	5.27E−08	1.41E−06	4.90E-06
	Adults	1.01E−10	5.19E−08	2.05E−07	2.47E-07	6.08E−09	1.91E−07	7.01E-07
HI	Children	1.15E−06	2.16E−04	2.67E−03	1.41E-03	2.11E−04	3.07E−02	3.52E−02
	Adults	1.17E-07	2.49E−05	3.10E−04	2.58E−05	2.47E-05	4.84E−04	8.70E−04
LADD	Adults	–	–	–	–	7.92E−08	1.27E−07	2.06E-07
CR	Adults	–	–	–	–	1.11E−09	2.42E−10	1.35E−09

The results of the average daily dose (ADDs) of six investigated PAEs via the three major exposure routes in indoor dust for children and adults are indicated in Table 7. PAEs total intake of ADDs (ADD_ing_ + ADD_der_ + ADD_inh_) from indoor dust for children and adults was found to be 3.37 × 10^–1^ and 1.70 × 10^–4^ mg/kg/d, respectively. This result implies that children are more susceptible to PAEs in indoor dust than adults. Children's ADDs from ingestion, dermal absorption, and inhalation of indoor dust were 9.84-, 6.31-, and 6.74- times higher than those of adults. This may be attributed to the frequent hand-to-mouth activities, increased exposure duration and children’s lower body weight. The ADDs of PAEs for children and adults via dust ingestion exposure were significantly higher than other exposure pathways in the study locations. The ADDs of PAEs for children and adults through dust ingestion were 1.46–1.56 orders higher than that of dust inhalation and dermal adsorption. Similar results have been reported and documented by other researchers and reviewers^12,76,78–80^.

The degree of PAEs exposure in adults was assessed via two approaches, the carcinogenic and the non-carcinogenic routes, as shown in Table 7. The findings revealed that the non-carcinogenic risks for adults and children were less than the stipulated limit of (HI > 1). Humans are considered exposed to non-cancer risks if the value of the hazard index (HI) is greater than 1. All the HI values were below one (1), indicating the absence of non-carcinogenic risk. However, the HI of DEHP (3.07 × 10^–2^), DBP (2.67 × 10^–3^) and D*n*OP (1.40 × 10^–3^) via indoor dust ingestion were close to the threshold value. Also, the hazard quotient of non-cancer risk for both children and adults for the six priority PAEs compounds in the sampled locations were in the ascending order of DEHP >  D*n*OP  > DBP > BBP > DEP > DMP. DEHP (3.07 $\times$ 10^−2^) and D*n*OP (2.58 × 10^–4^) contributed the most to the degree of hazard index among the nine study locations for children and adults, respectively.

Table 7 indicates that the carcinogenic risk for BBP and DEHP in indoor dust for adults through the three key predominant routes were 1.11 × 10^–9^ and 2.42 × 10^–10^, respectively. These values were below the carcinogenic threshold limits of 1.00 × 10^–4^ to 1.00 × 10^–6^ for PAEs exposure in humans. As a result, the carcinogenic risk of human exposure to BBP and DEHP in indoor dust was negligible. This is consistent with other reported research works on PAEs in indoor dust for BBP and DEHP, respectively: Li and Wang^61^ (9.30 × 10^–7^), (1.81 × 10^–4^); Li et al^12^ (1.30 × 10^–10^), (3.97 × 10^–7^); Abdi et al^11^(3.17 × 10^–10^), (2.45 × 10^–6^).

**Figure 4:**
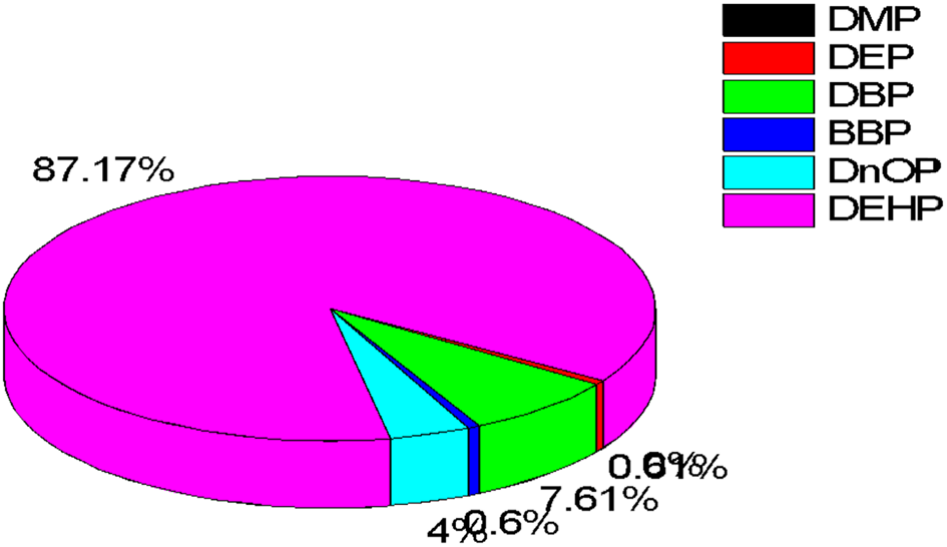
Percentage detection frequency of PAEs congener in indoor dust via ingestion route for children.

**Figure 5:**
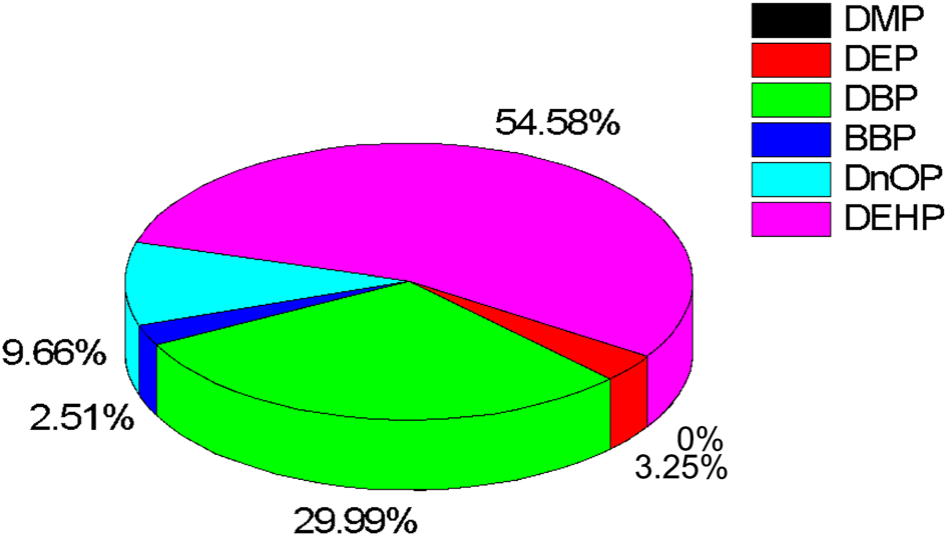
Percentage detection frequency of PAEs congener in indoor dust via ingestion route for adults.

However, the three predominant exposure routes for children were significantly higher than those of adults in both carcinogenic and non-carcinogenic risk estimation. This increase may be attributed to the explorative nature of children, their frequent hand-to-mouth activities, lower body weight and higher exposure duration. On the contrary, the dermal absorption and ingestion exposure routes were relatively similar and comparable for adults. This study’s results clearly highlight dust ingestion as the predominant route of PAEs in children and adults. Also, the total exposure of children to PAEs via ingestion route in indoor dust particles, as shown in Fig. 4, indicated that DEHP had the highest contribution of 87.2%, followed by DBP (7.61%) and D*n*OP (4.0%). DMP, DEP and BBP made an insignificant contribution of 0.01%, 1.23% and 0.60%, respectively. Similarly as shown in Fig. 5﻿ for adults, DEHP (54.6%) contributed the highest, followed by DBP (29.9%) and D*n*OP (9.7%). While DMP, DEP and BBP had 0.01%, 3.25%, and 2.51% contributions, respectively. This study’s results corroborate with other reported work^12,29,69^.

### Conclusion and recommendations

This study is the first to report the occurrence of six predominant PAEs compounds, namely D*n*OP, DBP, DEP, BBP, DMP and DEHP and their human health risk assessments using indoor dust as the environmental monitoring tool. Fifteen samples were collected from nine different indoor microenvironments in Ikeja, Lagos and Ota, Ogun States, Nigeria. D*n*OP was the most predominant contributor of PAEs, in indoor dust except for locations A, H and I, where DEP was the dominant contaminant. The mean levels of Σ_6_PAEs in indoor dust ranged from 1.61 ± 0.12 to 53.3 ± 5.27 μg/g across the different microenvironments. The highest level of Σ_6_PAEs in the microenvironments was in the order of location G (53.3 ± 5.27 μg/g) > location B (33.6 ± 3.10) and > F (15.3 ± 0.62 μg/g). The highest contributions of D*n*OP and DEP could be linked with plasticizers and personal care products containing D*n*OP and DEP, in addition to the contribution from flooring types, cleaning habits, and building materials in the indoor environment. The human health risk evaluation indicated ingestion of indoor dust as the dominant exposure route of PAEs for both children and adults, while the children were at a greater risk of PAEs. The non-carcinogenic risk exposure was below the threshold limit of (HI > 1), indicating no risk. Also, the result of the carcinogenic risk exposure of DEHP and BBP from indoor dust by adults was within the recommended threshold of 1.00 × 10^–4^ to 1.00 × 10^–6^. More attention should be paid to the indoor environment, and appropriate policies and procedures should be enacted by all stakeholders in order to reduce PAEs exposure to humans. A major limitation is the difficulty in obtaining the required permissions to access preschool centres and financial constrains in the analysis of samples thus resulting in the small sample size reported in this study. As a result, studies with a large pool of samples are required for a comprehensive understanding of the occurrence and distribution of PAEs in children’s microenvironments and the availability of research grants specifically for PAES analysis in children’s microenvironments is required to conduct a better comparative analysis of result in Africa. Also, future studies should include a comparative analysis of PAEs in both indoor and outdoor environments, source apportionment to ascertain the primary sources of PAE emissions and the environmental fate of PAEs in indoor environments. This will provide a scientific and robust understanding of the occurrence of PAEs in indoor environments.

## Supplementary Information

Supplementary Information.

## Data Availability

The data supporting the findings of this study are available on request from the corresponding author.
