# Supplementary material for: Levels and health risk assessments of Phthalate acid esters in indoor dust of some microenvironments within Ikeja and Ota, Nigeria
Source: Sci Rep. 2023 Jul 11;13:11209. doi: 10.1038/s41598-023-38062-4 (PMC10336085; doi:10.1038/s41598-023-38062-4)
Supplement: Supplementary file 1 — Supplementary Information. [file 41598_2023_38062_MOESM1_ESM.docx]

**Supplementary data**

**Levels and health risk assessments of phthalate acid esters in indoor dust of some microenvironments within Ikeja and Ota, Nigeria**

Winifred U Anake* and Esther A Nnamani

Department, of Chemistry, College of Science and Technology. Covenant University, Km 10 Idiroko Road, Ota, Nigeria

^*^Corresponding author: winifred.anake@covenantuniversity.edu.ng

ORCID ID:  0000-0002-8377-7629

# This SI file contains: 3 Pages

3 Tables

1 Figure

| S/No | Location | Sample location  code | Specific study  location | Flooring type | Frequently used ventilation type | No of plastic materials & furniture | Plastic toys |
| --- | --- | --- | --- | --- | --- | --- | --- |
| 1. | Ikeja, Lagos | A | Preschool | Cement | Fan and open windows | 5 | Yes |
| 2. | Ikeja, Lagos | B | Preschool | Cement | Fan and open windows | 45 | Yes |
| 3. | Ikeja, Lagos | C | Preschool | Broken tile | Fan and open windows | 12 | Yes |
| 4. | Ikeja, Lagos | D | Preschool | Terazzo | Air conditioner and open windows | 7 | Yes |
| 5. | Ikeja, Lagos | E | Preschool | Tiles | Fan and open windows | 5 | Yes |
| 6. | Ikeja, Lagos | F | Preschool | Polyvinyl  chloride carpet | Fan and open windows | 3 | Yes |
| 7. | Ota, Ogun | G | Hospital  (pediatric section) | Tiles | Air conditioner and open windows | 4 | No |
| 8. | Ota, Ogun | H | University hostel | Terazzo | Fan and open windows | 7 | No |
| 9 | Ota, Ogun | I | Universitychapel | Terazzo | Air conditioner and open windows | 4 | No |

Table S1: Overview of the study locations indoor characteristics and material

Table S2. Limit of detection and limit of quantification of the six PAEs congener

| PAEs congener | LOD | LOQ |
| --- | --- | --- |
| DMP | 0.007 | 0.020 |
| DEP | 0.005 | 0.016 |
| DBP | 0.025 | 0.083 |
| BBP | 0.005 | 0.018 |
| DnOP | 0.025 | 0.083 |
| DEHP | 0.014 | 0.046 |

Table S3. Concentrations of PAEs in Indoor Dust

| Sample code | Minimum | Maximum | Mean | Standard deviation |
| --- | --- | --- | --- | --- |
| A | 1.02 | 3.66 | 2.34 | 1.86 |
| B | 31.4 | 35.8 | 33.6 | 3.10 |
| C | 1.52 | 1.69 | 1.61 | 0.12 |
| D | 6.15 | 8.08 | 7.12 | 1.37 |
| E | 3.04 | 3.40 | 3.22 | 0.25 |
| F | 14.9 | 15.8 | 15.4 | 0.62 |
| G | 49.6 | 57.0 | 53.3 | 5.27 |
| H | 10.9 | 12.8 | 11.9 | 1.24 |
| I | 12.8 | 14.4 | 13.6 | 1.14 |


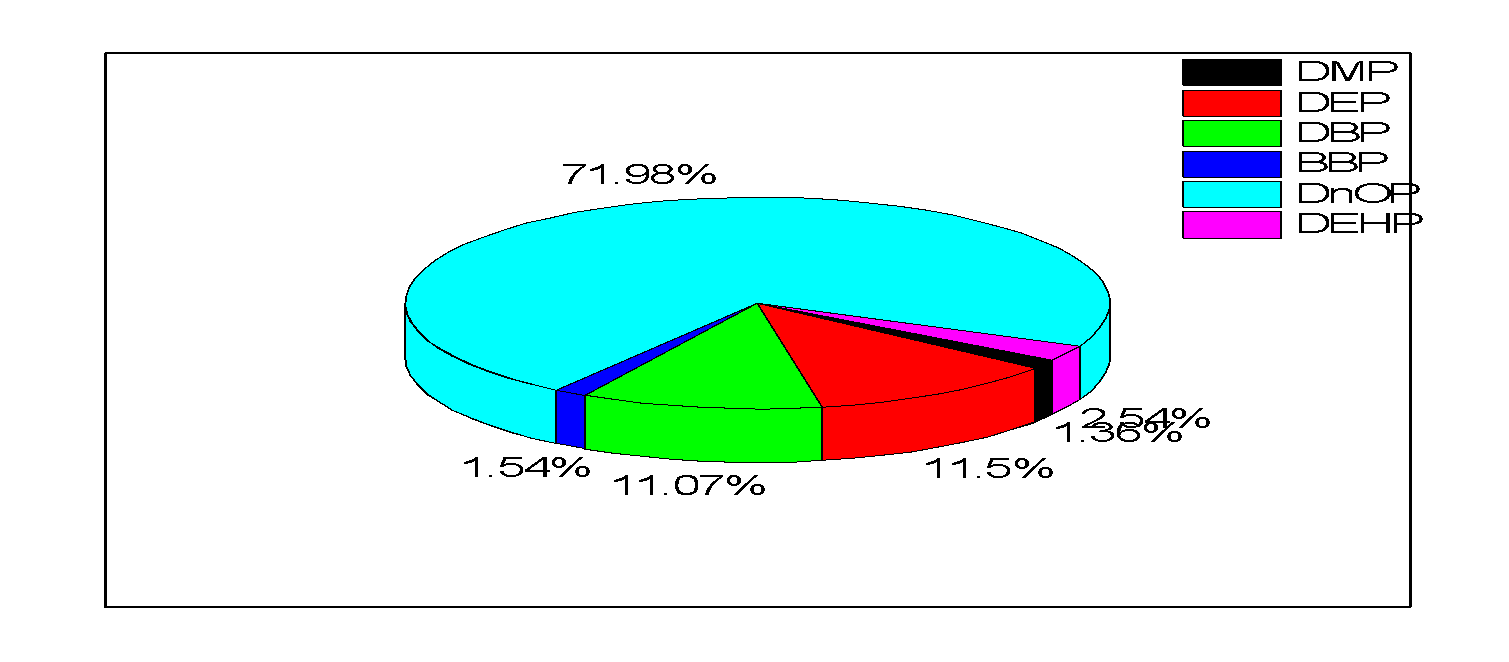


**Figure S1: Detection frequency of total PAEs compounds in indoor dust particles**
